# Exploiting Sentinel-2 dataset to assess flow intermittency in non-perennial rivers

**DOI:** 10.1038/s41598-022-26034-z

**Published:** 2022-12-16

**Authors:** Carmela Cavallo, Maria Nicolina Papa, Giovanni Negro, Massimiliano Gargiulo, Giuseppe Ruello, Paolo Vezza

**Affiliations:** 1 Department of Civil Engineering, University of Salerno, 84084 Fisciano, SA Italy; 2 Department of Environment, Land and Infrastructure Engineering, Polytechnic University of Torino, 10129 Torino, Italy; 3 Department of Information Technology and Electrical Engineering, University of Napoli “Federico II”, 80125 Napoli, Italy; 4 CIRA, Italian Aerospace Research Centre, 81043 Capua, Caserta, Italy

**Keywords:** Climate change, Hydrology, Engineering

## Abstract

Knowledge about the frequency and duration of each flowing status of non-perennial rivers is severely limited by the small number of streamflow gauges and reliable prediction of surface water presence by hydrological models. In this study, multispectral Sentinel-2 images were used to detect and monitor changes in water surface presence along three non-perennial Mediterranean rivers located in southern Italy. Examining the reflectance values of water, sediment and vegetation covers, the bands in which these classes are most differentiated were identified. It emerged that the false-color composition of the Sentinel-2 bands SWIR, NIR and RED allows water surfaces to be clearly distinguished from the other components of the river corridor. From the false-color composite images, it was possible to identify the three distinct flowing status of non-perennial rivers: “flowing” (F), “ponding” (P) and “dry” (D). The results were compared with field data and very high-resolution images. The flowing status was identified for all archive images not affected by cloud cover. The obtained dataset allowed to train Random Forest (RF) models able to fill temporal gaps between satellite images, and predict the occurrence of one of the three flowing status (F/P/D) on a daily scale. The most important predictors of the RF models were the cumulative rainfall and air temperature data before the date of satellite image acquisition. The performances of RF models were very high, with total accuracy of 0.82–0.97 and true skill statistic of 0.64–0.95. The annual non-flowing period (phases P and D) of the monitored rivers was assessed in range 5 to 192 days depending on the river reach. Due to the easy-to-use algorithm and the global, freely available satellite imagery, this innovative technique has large application potential to describe flowing status of non-perennial rivers and estimate frequency and duration of surface water presence.

## Introduction

Non-perennial rivers (NPRs) are ubiquitous watercourses which are characterized by the occurrence of non-flow periods, with dry stream beds or chains of isolated ponds of water. A recent study^1^ assessed that water ceases to flow for at least one day per year along more than half of the world’s river network. In addition, the extension of NPRs network is expected to increase in the near future because of anthropogenic pressures such as water withdrawals, land use change and climate change. These causes can turn perennial rivers into non-perennial ones or increase the non-flowing periods in streams which are already non-perennial^2^.

Due to the combination and succession of both terrestrial and aquatic ecosystems, the time-averaged biodiversity of non-perennial rivers is extremely high. These watercourses may support important ecosystem processes and provide valuable goods and services, such as the provision of materials (water and timber), freshwater and riparian biodiversity, regulation of biogeochemical cycles, and an ecological corridor for both wild and herded animals^3^. These highly dynamic environments require specific management framework to protect their ecological and socio-economic values^4^. In Europe, it is necessary to develop reliable methods and indicators for the assessment of “ecological status” for these types of water bodies, as required by the European Water Framework Directive (WFD 2000/60/EC-European Union Council^5^). However, non-perennial rivers are still not fully recognized in the WFD, in which they are a classified type of water bodies only in Mediterranean regions^6^.

One of the main obstacles for the implementation of correct management policies is the lack of information on the ecological functioning of non-perennial rivers. In particular, there is no adequate data about the duration and frequency of non-flow periods that are the primary determinants of ecosystem processes. River flow intermittence influence directly or indirectly biological communities, depending on the characteristics of low flow and non-flowing periods. The knowledge of flow intermittence patterns (frequency and duration of water presence at the reach scale) is essential for many applications such as the setting of environmental flow requirements, the evaluation of hydrological alteration, the estimation of the river capacity to assimilate potential contaminants and the optimized programming of monitoring plans^7^. The application of WFD 2000/60/EC^5^ requires the classification of ecological status based on comparison with a reference condition (i.e. a similar but undisturbed river). Consequently, it is necessary to know the degree of intermittence (undisturbed and possibly altered) in order to correctly apply the directive^8,9^.

Hydrological indicators used to measure flow intermittence patterns are estimated through sufficiently long time series of measured or simulated streamflow on a daily or monthly scale. For streams characterized by rapid flow regime changes, such as the small streams of Mediterranean-climate, daily data recorded over at least 20 years are often necessary^10^. However, streamflow gauges are rarely present in NPRs^1^ and have some criticisms in representing the spatial variability of areas submerged by water, especially during the low flow period and during the ponding phase, i.e. when the water in river channels is confined in isolated ponds^11^. Given the scarcity of direct discharge measures, hydrological models were proposed to simulate discharge time series and the regime over a long period of time^10^. However, the use of hydrological models may require a large amount of data to quantify the parameters governing the rainfall-runoff processes^12^. Another significant limitation is the lack of traditional gauging stations for calibrating the models^13^. In addition, many models have been developed with reference to perennial rivers and may be unsuitable for simulating the hydrologic regime of NPRs^14^. To simplify the problem, some authors have developed models to predict the intermittence class alone rather than the continuous series of flow rates over time. Snelder et al.^7^ used random forest models to relate intermittence classes to climate, catchment area, shape and slope. They attributed the model's poor performance to the fact that intermittency is also governed by other factors such as water table fluctuations and seepage through river bed. These processes, which are strongly heterogeneous, result in a low spatial correlation of intermittence patterns. Messager et al.^1^ developed a statistical random forest (RF) model to estimate the distribution of intermittent rivers and ephemeral streams across the globe. Observed streamflow data were linked to 113 potential predictors related to climate, physiography, land cover, soil, geology and groundwater.

It is important to state that knowledge of the flow rate (measured or simulated) in a specific section of a river is not sufficient to describe the space-pattern of presence/absence of water along entire river segments^15^. In the presence of strong spatial variability in the flow occurrence and surface water presence, the extrapolation of point measurements, recorded in one river cross-section, to ungauged ones may generate high uncertainty, even one has to predict presence/absence of surface water. Moreover, NPRs may be characterized by long non-flowing periods with presence of isolated pools and ponds. The spatial extension and duration of this ponding phase cannot be easily extrapolated from tradition ganging station and an overall view of the river reach is necessary to identify frequency and duration of such flowing status (namely ponding phase). This is possible with an airborne or ground photographic survey. However, due to the high costs, the spatial coverage and temporal resolution of these surveys are limited and therefore they lack in capturing the high spatio-temporal variability of NPRs' flowing status.

In this context, the use of satellite data constitutes a unique and crucial resource. Satellite data has the potential to cost-effectively monitor large areas (global coverage) with high temporal resolutions (in some cases daily or weekly acquisitions are available). Their use for monitoring the flowing status of NPRs has so far been limited by two factors: the spatial resolution of the satellite images and the availability of images at affordable costs. High resolutions are needed for monitoring small rivers, a characteristic often associated with the transient hydrological regime. Very high-resolution images (space resolution of the order of 0.5 m) are available for commercial use but their use for continuous monitoring in long time intervals is limited by the high costs of the products. Among freely distributed multispectral images with systematic global coverage, the Sentinel-2 mission provides the highest spatial resolution (10 or 20 m, depending on the band) and revisiting frequency (10 days, with one satellite in orbit since 2015 and 5 days with two satellite since 2017).

Satellite monitoring of river wet channels has received much attention in the recent scientific literature. Jiang et al.^16^ used Landsat TM (Thematic Mapper) and ETM (Enhanced Thematic Mapper Plus) images, with 30 m spatial resolution, for wet channel mapping of narrow (less than three pixels wide) and large rivers. Cavallo et al.^17^ used Landsat 4-5 and 8 to identify morphological changes in the Po River over a time period of approximately 30 years. Carbonneau et al.^18^ used Sentinel-2 images to delimit water, vegetation, and dry-sediment on various Italian rivers from 30 to 300 m wide. Seaton et al.^19^ detected changes in the size of water pools in non-perennial rivers with average width of 40-100 m in the Western Cape, South Africa, using a combination of Landsat 8 and Sentinel-2 datasets. Hou et al.^20^ employed the archive of Landsat-5 and Landsat-7 to estimate a parameter that describes the shape of the relationship between wet channel width and its frequency of occurrence, for more than one million river reaches in Australia. This parameter was used to classify the degree of intermittency as a function of the frequency with which the river width is close to its maximum or minimum value.

In this study we developed a procedure to estimate the duration of three different flowing status (flowing, ponding and dry phases) in NPRs. To do this we processed Sentinel-2 data to generate false color images (FCIs) in which the water pixels stand out from the background. Field data and very high-resolution images acquired in various conditions of water flow were used to evaluate the reliability and operational limits of satellite observations. Three different flowing status were identified for all suitable and available archive images (seven years of observation). This information was used to calibrate Random Forest (RF) models able to predict the daily occurrence of each flowing status at the river reach scale, using precipitation and air temperature data as input variables. In particular, two types of binary RF models were developed, the first was implemented to differentiate between flowing and non-flowing (ponding and dry) phases and the second further detailed the non-flowing phase by discriminating between ponding and dry phases. The analysis was developed for five morphologically homogeneous river reaches belonging to three small streams in the Campania region (Southern Italy): Sciarapotamo, Mingardo and Lambro.

## Case study

**Figure 1:**
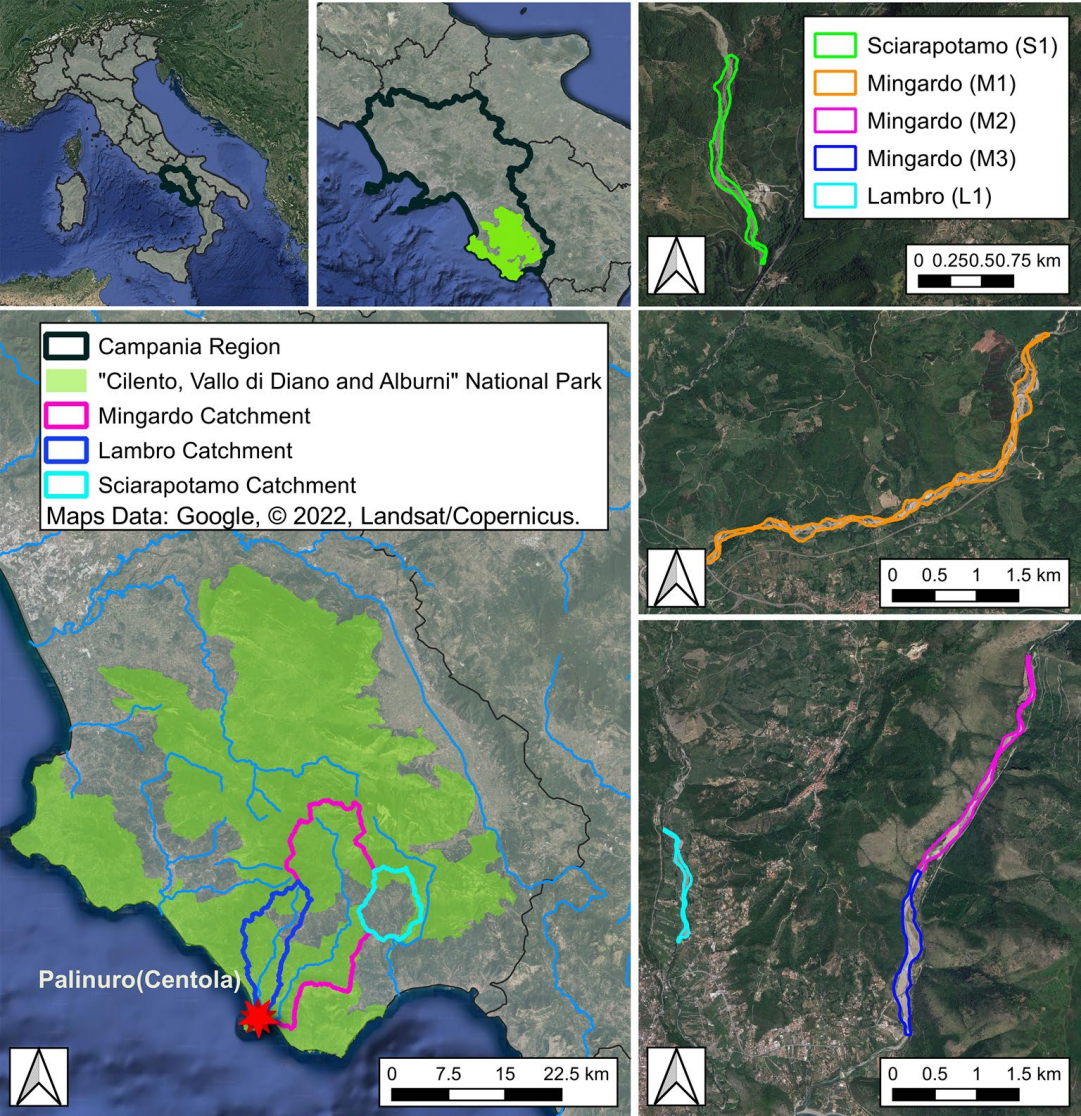
Geographical framework of the study area. Maps Data: Google, © 2022, Landsat/Copernicus. QGIS 3.10.11 https://www.qgis.org/it/site/forusers/download.html.

The study cases are five reaches of the river network of the “Cilento, Vallo di Diano and Alburni” National Park, located in the province of Salerno, in the Campania region (Southern Italy). The National Park covers an area of 1810 km^2^, stretching from the Tyrrhenian coast to the foot of the Apennines (see Fig. 1), with an altitude range from sea level to the top of the Cervati Mountain, at 1898 m a.s.l.. Established in 1991 to preserve its great flora and fauna biodiversity, it is one of the widest Italian National Park. It includes 29 Sites of Community Interest (SIC) and 8 Special Protection Areas (SPA), set in application of the European Habitat (92/43 / EEC) and Birds (79/409 / EEC) Directives. Fauna species of considerable community importance are present, such as the otter (*Lutra lutra*), that is the icon symbol of the park, many amphibians (e.g. *Bombina pachipus* and *Salamandrina terdigitata*), odonates (*Oxygastra curtisii* and *Coenagrion mercuriale*) and fish (*Lampetra planeri* and *Rutilus rubilio*). From a geological point of view the Park is mainly composed of carbonate and terrigenous mountain massifs, marly-clayey hills as well as alluvial and coastal plains^21^. A system of carbonate aquifers produces a complex underground circulation and the presence of several springs. The climate is characterized by a wet season from September to May, the rainiest month is November with an average of around 90 mm, the driest month is July with an average of around 10 mm. The total annual average is around 1553 mm^22^. While, considering only the period of observation (2015–2021) it was on average 1300 mm. The warm season June–September has a maximum daily temperature above 27 °C, and the cool season November-March has a maximum daily temperature below 17 °C. Five morphologically homogeneous river reaches were investigated: three reaches (M1, M2, M3) belong to the Mingardo, one (L1) to the Lambro and one (S1) to the Sciarapotamo streams (see Fig. 1).

In Table 1 some significant characteristics of the studied reaches are reported. The drained catchments were calculated by placing the closure section at the downstream end of each reach, the average width was calculated from the ratio of the area of the active channel to the length of the center line. River reach morphology was evaluated following the approach proposed by Gurnell et al.^23^.

**Table 1:** Characteristics of the studied reaches.

Catchment	Reach	Drained catchment [km^2^]	Average width [m]	Reach morphology
Sciarapotamo	S1	54	107	PC-W
Mingardo	M1	180	77	PC-W
Mingardo	M2	221	62	C-S
Mingardo	M3	225	117	PC-W
Lambro	L1	74	38	U-W

Reach morphology codes: C = confined; PC = partially confined; U = unconfined; W = wandering; S = sinuous.

### Sciarapotamo River

The Sciarapotamo River is a right tributary of the Bussento River, it has a catchment area of about 54 km^2^ and a length of about 11 km. The study reach (S1) ends at about 500 m upstream of the confluence with the Bussento River, (Fig. 1), it has a narrow channel with a wandering planform (braiding index = 1.21, and sinuosity index = 1.09^23^). The River Basin Management Plan (RBMP)^24^, developed by the Southern Apennines Basin Authority (Autorità di Bacino Distrettuale dell’Appennino Merdionale), classified the Sciarapotamo as intermittent. It has only one withdrawal from groundwater and with reference to the WFD 2000/60/EC, it is deemed to have high ecological status and good chemical status.

### Mingardo River

The Mingardo River is characterized by the largest drainage area (about 232 km^2^). It originates in the Gelbison mountain and flows in the Tirreno sea close to the village of Palinuro with a total length of 38 km. Three reaches are investigated, the upstream wandering channel (M1) and two other consecutive reaches closer to the outlet, the upstream of these two is a single-thread sinuous reach (M2) and the subsequent one is a wandering reach (M3). The main outlet of the floodplain aquifer is into the river, around 600 m downstream the end of reach M3. According to the RBMP^24^ all the studied reaches of the Mingardo are perennial, there are withdrawals from groundwater and springs, and the ecological and chemical status are good^5^ for all the reaches.

### Lambro River

The Lambro River, with a total length of 22 km and a drained catchment of about 78 km^2^, originates in the Gelbison mountain and flows into the Tirreno sea about 500 m north of the mouth of the Mingardo River. The studied reach (L1) is wandering. According to the RBMP^24^ the Lambro is perennial, there are withdrawals from springs for both domestic use and irrigation. The ecological status is sufficient and so is the chemical status^5^.

## Materials and methods

### Remote sensing datasets and field surveys

The choice of the most suitable sensor to observe a specific physical process depends on many factors^25^. One of the most important aspects is the definition of the needed spatial resolution of satellite data that depends on the dimension of the target. Following Jiang et al.^16^ the minimum width of the river that can be monitored is equal to three image pixels. When the objective is to monitor the flowing status of non-perennial rivers, the width of the wet channel is also particularly relevant. Another critical issue is represented by the temporal resolution, which depends on the speed of evolution of the observed process^26^. A weekly revisit frequency may be not sufficient in many cases, because the flooding or drying process of the river can be much quicker. Moreover, to fully describe frequency and duration of NPR flowing status, a multi-year archive of data must be available. Given the large number of required images, the cost of imagery acquisition can become a limiting factor. Among the multispectral images freely distributed with systematic global coverage, the Sentinel-2 mission provides the highest spatial resolution and revisit frequency. Sentinel-2 is a mission of the European Space Agency (ESA) under the Copernicus program. It comprises a constellation of two identical satellites, Sentinel-2A and Sentinel-2B launched on 23 June 2015 and 17 March 2017, respectively. With both satellites in orbit, in the studied area the revisit frequency is 5 days. Both satellites carry on board the Multispectral Instrument (MSI), which provides 13 spectral bands in the visible, near infrared (NIR) and short wave infrared (SWIR) wavelengths, with four bands at 10 m (B2, B3, B4, B8), six bands at 20 m (B5, B6, B7, B8a, B11, B12), and three bands at 60 m (B1, B9, B10). In Table 2, the spectral characteristics of Sentinel-2 data are shown.

**Table 2:** Characteristics of Sentinel-2 MSI.

Wavelenghth range [nm]	Spatial resolution [m]			Spectral region
	10	20	60	
423–463			B1	Coastal aerosol
458–523	B2			Blue
543–578	B3			Green
650–680	B4			Red
698–713		B5		Red Edge
733–748		B6		Red Edge
773–793		B7		Red Edge
785–899	B8			NIR
855–875		B8a		NIR narrow
925–965			B9	Water-Vapour
1350–1410			B10	SWIR-Cirrus
1565–1655		B11		SWIR
2100–2280		B12		SWIR

Each Sentinel-2 image is a tile of 100 km × 100 km. Given the limited spatial extension of the selected river basins, all the analyzed river reaches were included within the same image. For the present study, we downloaded 141 images covering the years from 2015 to present from the Copernicus Open Access Hub (https://scihub.copernicus.eu/dhus/#/, last accessed on 8 December 2021). Level-2A Bottom-of-Atmosphere (BOA) images are available for most of the dataset, with the exception of images acquired in the early years of mission operation; for which an atmospheric correction with the Sen2cor tool of Sentinel Application Platform (SNAP) was performed. The lower resolution bands were all resampled to a resolution of 10 m using the bilinear interpolation provided by SNAP tool.

A very high-resolution satellite image (VHR), an orthophoto and field observations were then used as ground truths. The VHR image used is the one provided free of charge by Google Earth Pro, dating on 14 June 2019. The orthophoto was produced through a survey with a UAV (Unmanned Aerial Vehicle) performed on 13 December 2021. The UAV mounted a Hasselblad L1D-20c camera with exclusive Hasselblad Natural Colour Solution (HNCS) technology. The restitution and ortho-rectification of the acquired images were performed in Agisoft Metashape environment. Geo-located ground pictures and measurements of the width and depth of water ponds were taken during field surveys performed on 26 June 2020, 26 July 2020, 19 September 2020 and 6 February 2021.

### Meteo-hydrological data

The meteo-hydrological data were extracted from the “Centro Funzionale Multirischi della Protezione Civile Regione Campania” website (http://centrofunzionale.regione.campania.it/) covering the period 2015-2021. We used daily time series of rainfall, air temperature (maximum, mean, minimum) and water levels. The available measuring stations within or adjacent to the Sciarapotamo, Lambro and Mingardo catchments were respectively 6, 3 and 8 for rainfall and 3, 5 and 3 for temperature. Rainfall and temperature data were spatially interpolated on catchments area using Thiessen polygons method. Daily water levels were available at two gauging stations, located 500 m downstream L1 and M3 reaches respectively.

### Method for the identification of the flowing status

To distinguish as best as possible wet channel, sand and vegetated bars, the difference between spectral signatures of water, sediment and vegetation can be exploited^27^. Different spectral signatures are due to the fact that the reflected and emitted quantity of electromagnetic energy differs as the wavelength and the land usage conditions vary. Consequently, starting from the VHR image provided by Google Earth Pro, the geo-located pictures and the orthophoto derived by the UAV survey, we extracted several polygons with known land covers (water, bare sediments, grass and bushes). We plotted the spectral signatures across the Sentinel-2 bands, for the different soil cover classes, using the pixels contained within their respective polygons. All the Sentinel-2 bands were used except the atmospheric bands B1, B9 and B10. To account for the difference between seasonal variations of the flowing status, the spectral signatures were extracted in winter (February 2021), late spring (June 2019) and late autumn (December 2021). In the cases where the Google Earth Pro image (June 2019) and field observations (February 2021) were used as ground truth we considered four cover classes: water, bare sediments, grass and bushes. Whereas, in the case in which the more resolute, UAV acquired, orthophoto was available (December 2021), it was possible to further differentiate the areas with deeper water from those with shallower water.

A set of three bands was identified in which the three land covers water, sediment, and vegetation are most distinguishable. The three bands were overlaid to create false-color images (FCIs). The FCIs were then used to identify the flowing status on the day of the satellite acquisition. Specifically three flowing status were distinguished: 1. the longitudinal flow of water is continuous, this condition was named “flowing” (F); 2. there is no continuous flow, but a pattern of disconnected water ponds is present, this condition was named “ponding” (P), and 3. the riverbed is completely dry, or the water ponds are too small to be detected, this condition was named “dry” (D). The identification of the three flowing status was performed by visual interpretation of the FCIs. We inspected the FCIs at a fixed mapping scale of 1:20,000 to obtain a standardized result. Possible interpretation ambiguities were minimized by using three simple and well recognizable categories (flowing, ponding and drying) and by having the same interpreter perform the visual inspection on all of the imagery for consistency. Examples of labeled FCIs and a table of observed flowing status for all the images are reported in the supplementary materials (see Supplementary Information 1).

The flowing status identification is easily reproducible by anyone using the free Sentinel-2 dataset and the FCI_maker code that we developed and made available (https://code.earthengine.google.com/220275e2e31d308773432fdecadc8a2f). The FCI_maker code allows to visualize the FCIs images, it works in the Google Earth Engine (GEE) that is a freely available cloud computing portal, containing data collection of satellite imagery with global cover^28^. A step-by-step guide for the use of the FCI_maker code is provided at the following link (see Supplementary Information 2).

### Random forest classification

The Random Forest^29^ classification algorithm, as implemented in R^30^, was used to explore the relationship between the meteo-hydrological data (rainfall and air temperature) and the flowing status (F/P/D) of non-perennial rivers. The classification algorithm was trained using as predicted feature the classified flowing status, while the predictor features were represented by the rainfall and temperature data spatially interpolated at the catchment scale by Thiessen polygons. For this analysis, the water level time series were not taken into account as they were available only nearby reach M3 and L1. As the hydrological regime of non-perennial rivers can be disassembled into one flowing phase (F) and two non-flowing phases (P and D phases^31^), we developed binary classification RF models able to distinguish between flowing/non-flowing (F/NF) phases and the ponding/dry (P/D) phases. In this way for permanent ponding reaches (river stretches that never achieve a totally dry condition and wetted areas persist over time among subsequent flowing phases) exclusively one F/NF model was calibrated, whereas for drying reaches (river stretches that can experience the total disappearance of water table during non-flowing period) both F/NF and P/D models were applied. RF models were developed both at the local (individual reach models) and regional scale (all reaches together, Global Model-GM) and all model performances were evaluated. To address potential bias in the RF models due to class imbalance, we developed classification models using a random oversampling of the training dataset, by randomly sampling with replacement the observations from the minority class in both F/NF and P/D dataset (see e.g.^1^). As for reaches M1 and M2 a total dry phase was never assessed from the image analysis therefore exclusively the F/NF models were calibrated. In total 10 RF classification models were developed and tested.

As primary predictor variables of NPRs flowing status, we considered (i) the daily time series of rainfall (R) and (ii) maximum, mean and minimum air temperature (T_MAX, T_MEAN, T_MIN) from 2015 to 2021. To explore the effect of rainfall and temperature at different temporal scales, we aggregated the daily records into cumulative rainfall (R) variables and average temperature variables (T_MAX, T_MEAN, T_MIN) before the satellite image acquisition. For cumulative and average variables we considered 6 different time intervals, i.e., 3-5-7-10-30-90 days. A total of 28 predictor variables were therefore included in the RF analysis, using the following codes: R3, R5, R7, R10, R30 and R90 for rainfall variables, and T3_, T5_, T7_, T10_, T30_, T90_MAX, MEAN, MIN for air temperature variables.

To optimize the predictive performances of each RF model, we minimized the out-of-bag error (E_OOB_) carrying out variables selection and hyperparameters tuning. To reduce the number of predictors in RF models, we tried to select the smallest number of variables providing the best possible classification result (parsimonious model). Variables selection was performed by (i) using a common algorithm for features selection named Boruta^32^ and (ii) avoiding high correlation (Spearman’s rho correlation coefficient > 0.7) between selected variables^33^. The Boruta algorithm is implemented in R as a package and allows the user to identify the most important predictors for classification purposes. It relied on the random forest classification algorithm and, through an iterative procedure, the most significative predictors are identified by comparing the relevance of these features with a randomized version of them. Three hyperparameters were tuned^33–35^: (i) the number of decision trees (*ntree*) defined as two times the replicates required to stabilize the E_OOB_, (ii) the number of variables randomly sampled in each node (*mtry*) computed as the square root of the total number of predictor features considered in each model (with a minimum of 2), and (iii) the amount of observations used to train each tree (*sampsize*) to maximize models performance. Predictive performances of developed RF models were assessed using four common performance metrics, i.e. accuracy, sensitivity, specificity, and true skill statistic (TSS^33,36^). Finally, individuals RF models were used to predict the daily occurrence of the flowing status in all considered reaches when the Sentinel-2 images were not available or exploitable during the period 2015–2021.

## Results

### Spectral signature analysis and false color images

**Figure 2:**
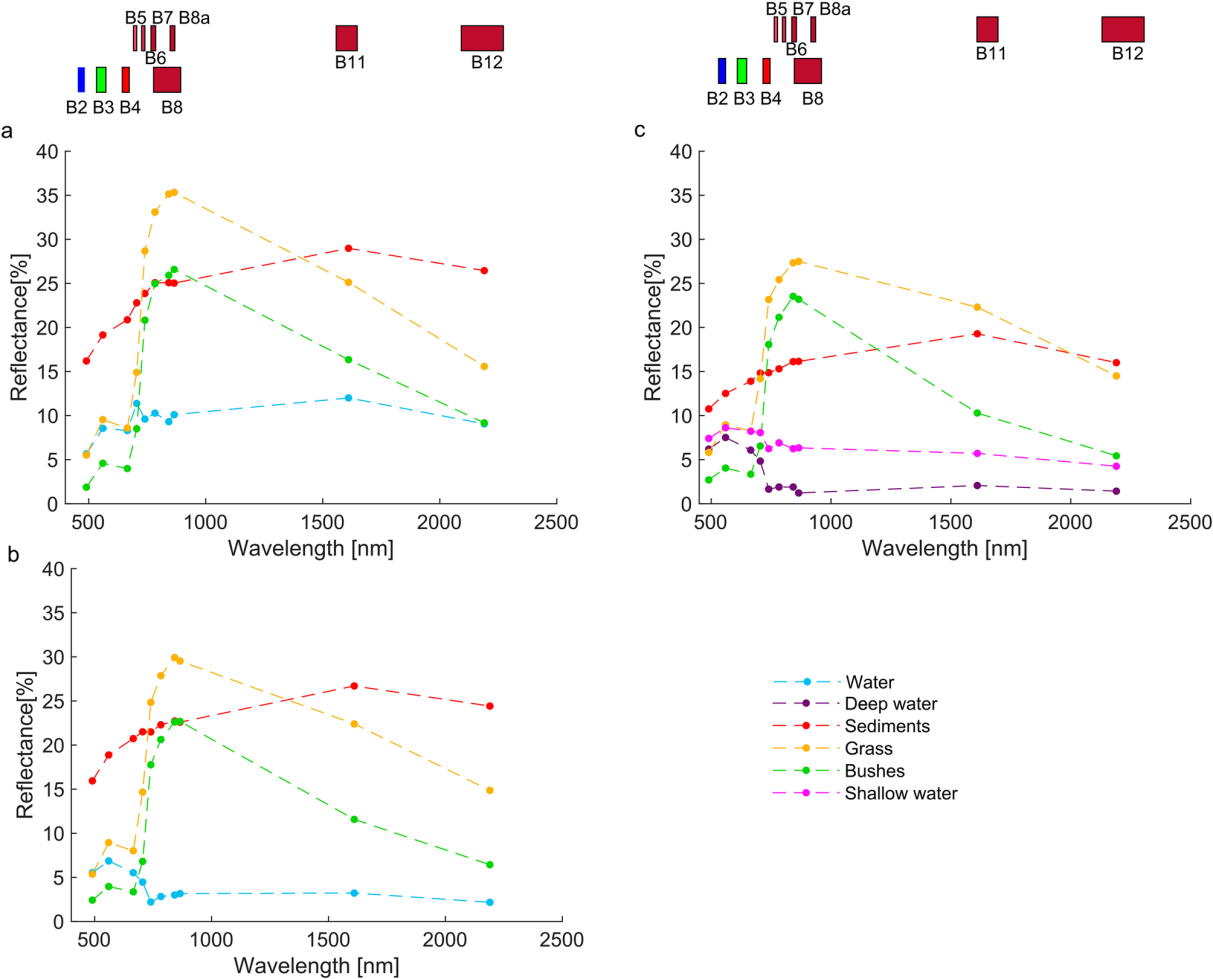
(**a**) Spectral signature of late spring season, Google Earth of June 2019, (**b**) spectral signature of winter, field pictures of February 2021, (**c**) spectral signature of late autumn season, ortophoto of December 2021.

The spectral signatures of the various analyzed land covers are shown in Fig. 2a–c. It can be seen that the reflectance values in the infrared bands are higher for shallow water than for deep water. This may be due to the fact that when the water depth is shallow the reflectance is affected by the response of the underlying sediment bed. The sediment curve of Fig. 2c is lower than the ones in Fig. 2a and b. This could be linked to the variation in water content, in fact at higher water content, the reflectance of sediment is known to be smaller in all wavelengths^37^. This explanation seems confirmed by the observation that the number of dry days before the acquisitions of June (Fig. 2a), February (Fig. 2b) and December (Fig. 2c) was 10, 4 and 1 respectively.

We can observe that the classes water, grass and bushes are little distinguishable in RGB images, because the red (B4), green (B3) and blue (B2) have very similar reflectance values. However, these bands allow to easily distinguish the mentioned classes from the sediments. In NIR bands, the reflectance is more variable between different land covers. For example, in B8 water and grass reflectance values are very different while sediment and bushes are still indistinguishable from each other.

**Figure 3:**
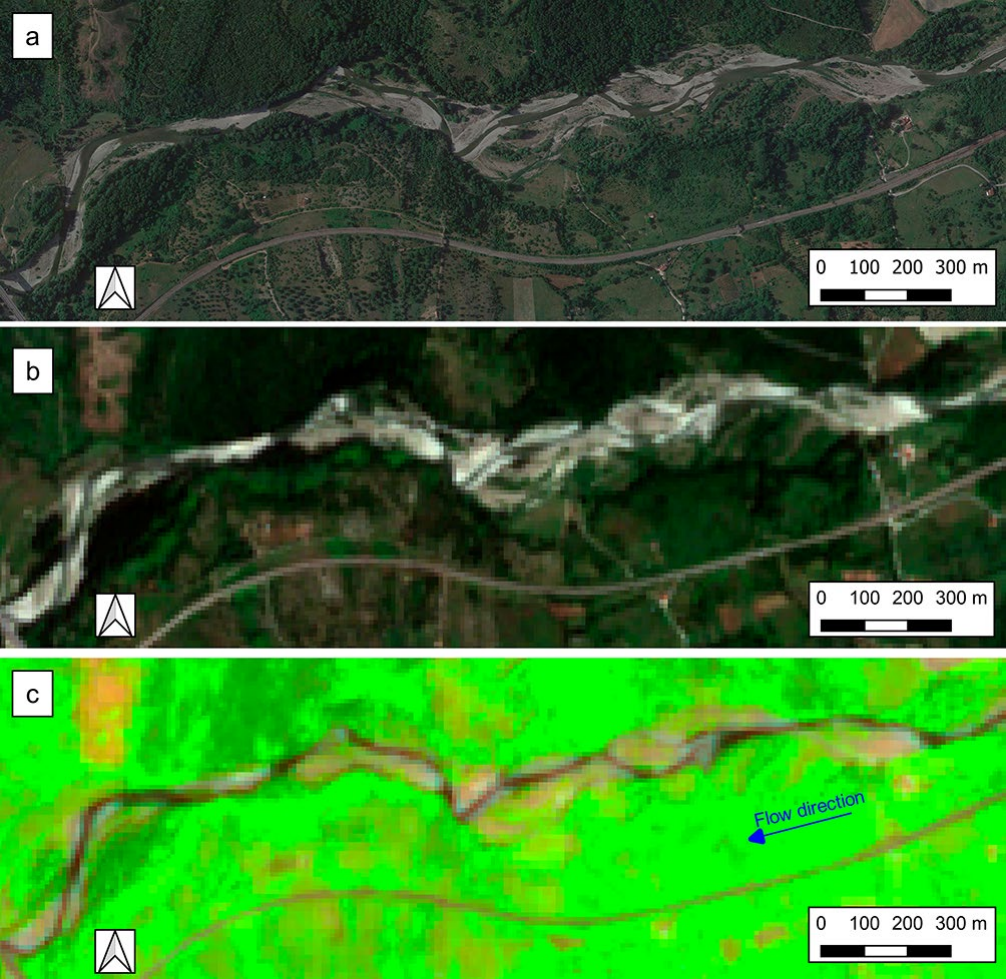
Test case n°1, M1 reach, (**a**) Maps Data: Google, © 14 June 2019, (**b**) RGB extracted by Sentinel-2 acquisition of 12 June 2019 and (**c**) FCI extracted by Sentinel-2 acquisition of 12 June 2019. QGIS 3.10.11 https://www.qgis.org/it/site/forusers/download.html.

**Figure 4:**
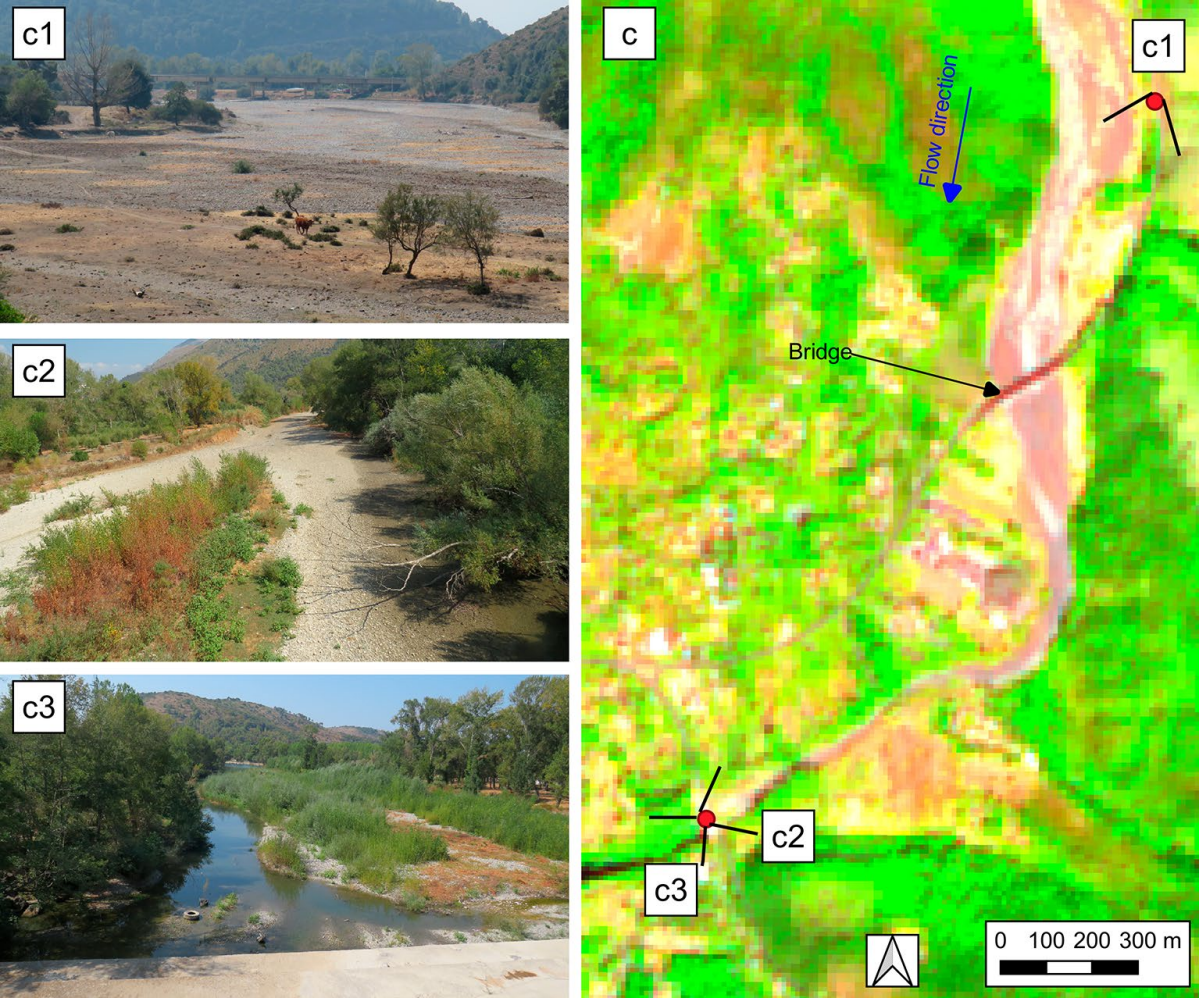
Test case n° 4, M3 reach, (**c**) FCI extracted by Sentinel-2 image of 19 September 2020 with the geolocation points of the photos, (**c1**), (**c2**) and (**c3**) geo-located photos take on 19 September 2020. QGIS 3.10.11 https://www.qgis.org/it/site/forusers/download.html.

In the SWIR, both B11 and B12 allow to distinguish fairly well all the classes. Unfortunately, these two bands have a coarser spatial resolution (20 m) than visible and B8 bands which are provided at 10 m. Since RGB images do not allow to differentiate adequately the land cover classes (see Fig. 3b), it is more convenient to create FCIs through the composition of 3 bands to allow better distinction between classes. An optimal composition was obtained with the bands B11 of the SWIR, the B8 of the NIR and the B4 of the visible. Despite the good capabilities of the SWIR bands for class separation, we used only one of the two, due to their coarser resolution. The B11 was finally chosen because it has higher capability of distinguishing between grass and water in summer season. Examples of FCIs obtained by the composition of bands 11, 8 and 4 and RGB are reported in Fig. 3c and b. In the FCIs composition, the color red was associated with B11, green with B8, and blue with B4. Consequently, the water has a black or dark blue color, while the vegetation has a green color with decreasing intensity, in some cases almost tending to yellow, as it passes from thick vegetation to sparse vegetation. The light pink color, on the other hand, is associated with sediments or soil without vegetation cover. In some situations, e.g. dry or large sediments, the sediments can also take on a white color (see Fig. 4c). It can be seen that in the FCIs the presence of water can be distinguished much better than in the RGB image (see Fig. 3b and c).

### Comparison of false color images with ground truth

To assess the actual possibility of observing the flowing status from the FCIs analysis, several comparisons were made with the ground truths introduced in paragraph “Remote sensing datasets and field surveys”. The test case’s comparisons are summarized in Table 3. It is worth noting that the field surveys and the orthophoto are contemporary with the Sentinel-2 acquisitions while the Google Earth Pro image was acquired two days after the coupled Sentinel-2 image. In the two-day interval between the two acquisitions, there was no precipitation, so the only possible changes were due to evaporation and consequent possible reduction in water surface area. However, since this was a very short period it is reasonable to assume that there was no significant change in the extension of the wet channel. The set of ground truths used provided a fairly good description of the various seasonal flowing status.

**Table 3:** Pairs of Sentinel-2 and ground truth images.

Test case	Ground truth source	Ground truth date	Sentinel-2 date	Study Reach
1	Google Earth Pro	14 June 2019	12 June 2019	M1, M2, M3, L1
2	Field survey	26 June 2020	26 June 2020	S1
3	Field survey	26 July 2020	26 July 2020	S1
4	Field survey	19 September 2020	19 September 2020	S1, M3
5	Field survey	6 February 2021	6 February 2021	M1, M3, S1
6	UAV	13 December 2021	13 December 2021	M3, S1

For the sake of brevity, only a selection of the performed comparisons is shown below. Figure 3 shows the test case n°1 for the M1 reach. It can be noticed that the wet channel clearly stands out from the other component of the river corridor in the FCIs images (Fig. 3c). On the contrary the wet channel is not easily distinguishable in the RGB (Fig. 3b) obtained by the same Sentinel-2 acquisition. Despite the much coarser resolution of the FCI (Fig. 3c) compared to the VHR (Fig. 3a), the wet channel is about equally visible and the continuous flow phase (F) can be clearly identified.

Figure 4 shows the test case n° 4 for M3 reach and for a section downstream of it. In this date the upstream section is completely dry as confirmed by the geo-localized photo c1. In the downstream part, in correspondence of the photo c2, it is possible to notice, both in the FCI and in the ground photo, an elongated pond in correspondence of the left bank of the riverbed. Immediately downstream, in the section framed by photo c3, a wet channel can be seen in the FCI which is confirmed in the ground photo. It can be seen that the FCI allows for a clear distinction between the different flowing status that occur along the riverbed. A possible source of misclassification is the presence of the bridge, which in this case takes on a color similar to water. However, the location of infrastructure can be usually established from other sources.

**Figure 5:**
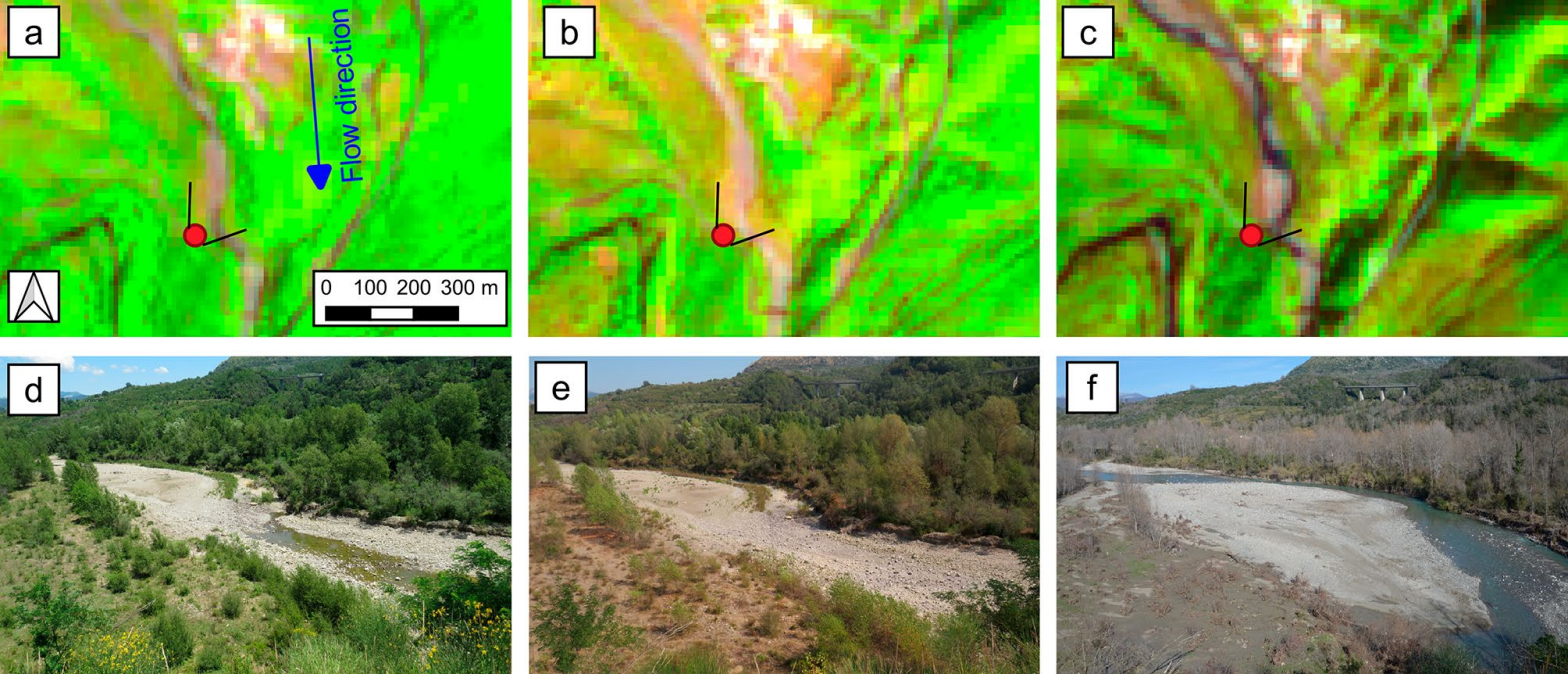
Test cases 2, 4, 5, S1 reach, (**a**) FCI of 26 June 2020, (**b**) FCI of 19 September 2020, (**c**) FCI of 6 February 2021, (red point) photo shooting location, (**d**) photo taken on 26 June 2020, (**e**) photo taken on 19 September 2020, and (**f**) photo taken on 6 February 2021. QGIS 3.10.11 https://www.qgis.org/it/site/forusers/download.html.

Figure 5 shows the test cases n° 2 (June 2020), n° 4 (September 2020) and n° 5 (February 2021) for S1 reach. The FCIs of Fig. 5a–c and the corresponding ground photos of Fig. 5d, e and f show the temporal evolution of the water presence. In June 2020 the FCI shows a P phase which is confirmed by the ground photo. In particular, the pond visible from the photo (Fig. 5d) is recognizable in the FCI (Fig. 5a) despite the very small size. In September 2020 at the end of the summer dry season, the bed is completely dry (Fig. 5b and e). In the FCI of February 2021 (Fig. 5c) a continuous flow line (F phase) is visible, as confirmed by the contemporaneous surveys (Fig. 5f). Figure 5d-f were taken from a point on the adjacent street to the S1 reach.

**Figure 6:**
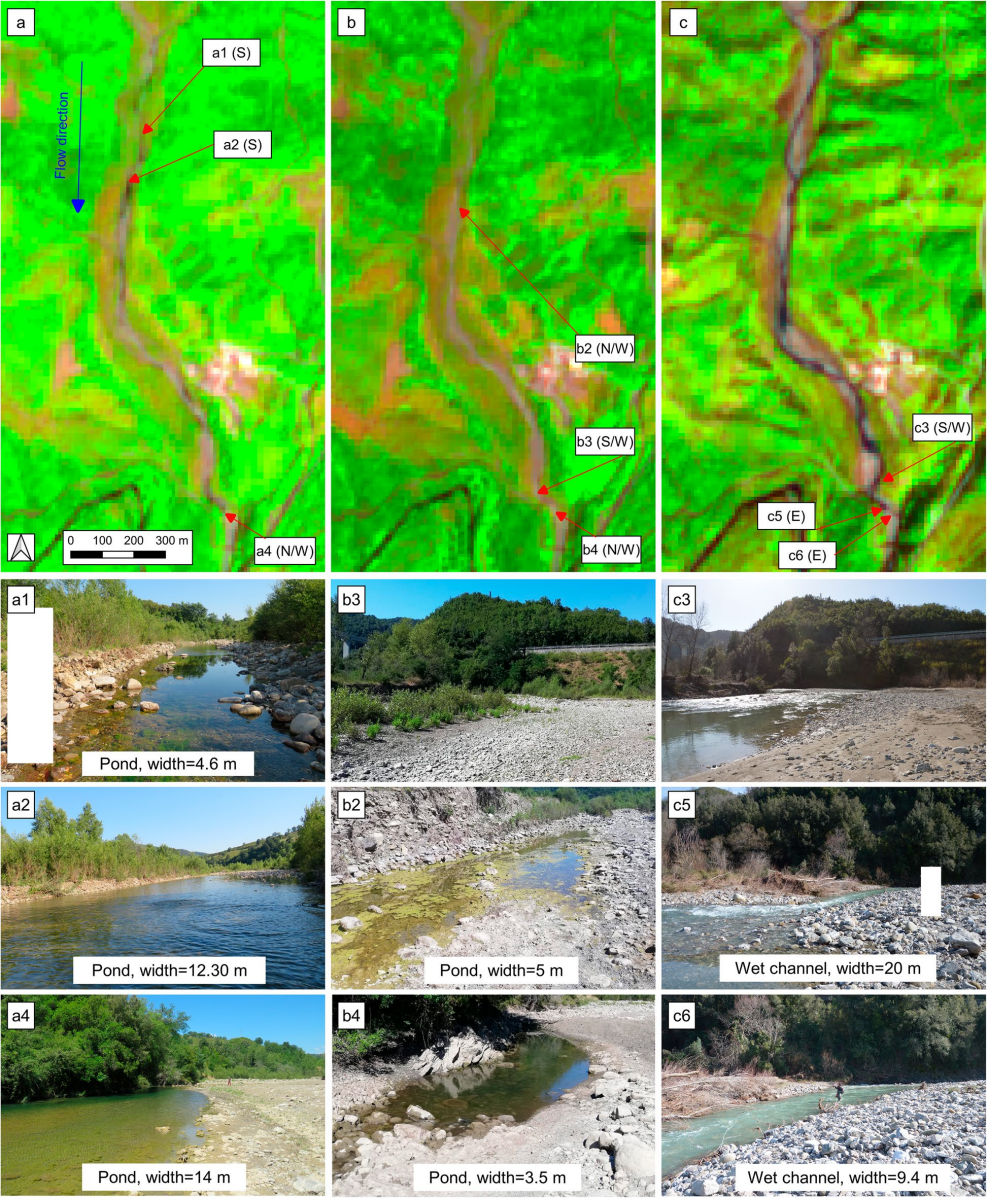
Test cases 2, 3, 5, S1 reach, (**a**) FCI of 26 June 2020, (**b**) FCI of 26 July 2020, (**c**) FCI of 6 February 2021, (a1,a2 and a4) photos taken on 26 June 2020, (b3,b2 and b4) photos taken on 26 July 2020, and (c3,c5 and c6) photos taken on 6 February 2021. Photo-taking orientation are reported in brackets in the labels of a, b and c panels. Note: White boxes are used to cover people incidentally taken from the photos. QGIS 3.10.11 https://www.qgis.org/it/site/forusers/download.html.

Figure 6 shows the test cases n° 2 (June 2020), n° 3 (July 2020) and n° 5 (February 2021) for S1 reach. Figure 6a1 shows the photo taken in correspondence of a pond with a length greater than 50 m and a width less than 5 m, the reduced size in width means that the pond is not clearly recognizable in the FCI of 26 June 2020 (Fig. 6a). The ponds wider than 10 m, shown in Fig. 6a2 and a4, are clearly visible in the FCI of 26 June 2020 (Fig. 6a). Figure 6b2 and b4 show the photos acquired on 26 July 2020, taken in correspondence of the same points represented in Fig. 6a2 and a4, in the month of July (see Fig. 6b2 and b4) the ponds were almost dry and are not visible in the FCI. Probably the reduction of the ponds is due to the low monthly rainfall (44.2 mm) and average monthly temperature (24 °C) occurred during the month. Figure 6b3 and c3 show images taken from the same point, where Fig. 6b3 shows a D phase correctly identified in Fig. 6b. Figure 6c3 shows a well recognizable F phase in FCI. Finally, Fig. 6c5 shows a photo taken in correspondence of the wet channel with a width of 20 m, while in Fig. 6c6 is showed the photo taken in correspondence of the wet channel with a width of about 9 m. From Fig. 6c, where the channel has a width of 20 m (Fig. 6c5), it is possible to identify the presence of water in FCI (Fig. 6c), while, when the width of the wet channel is reduced (Fig. 6c6), and is equal to 9.4 m, it is not possible to clearly distinguish the presence of water in FCI (Fig. 6c). The analysis described in this paragraph showed that the minimum width of ponds identifiable by FCI is highly variable. In some cases, it is even possible to identify ponds with widths between 6 and 10 m. In other conditions, however, ponds or wet channels larger than 10 m and less than 15 m are not clearly identifiable. This depends on the relative position of the object with respect to the pixels. In fact, pixel-size water surface contained in only one pixel is identified, whereas if it covers, only partially, two or more pixels the identification is more complicated.

### Relation between flowing status extracted by FCIs and hydrological data

**Figure 7:**
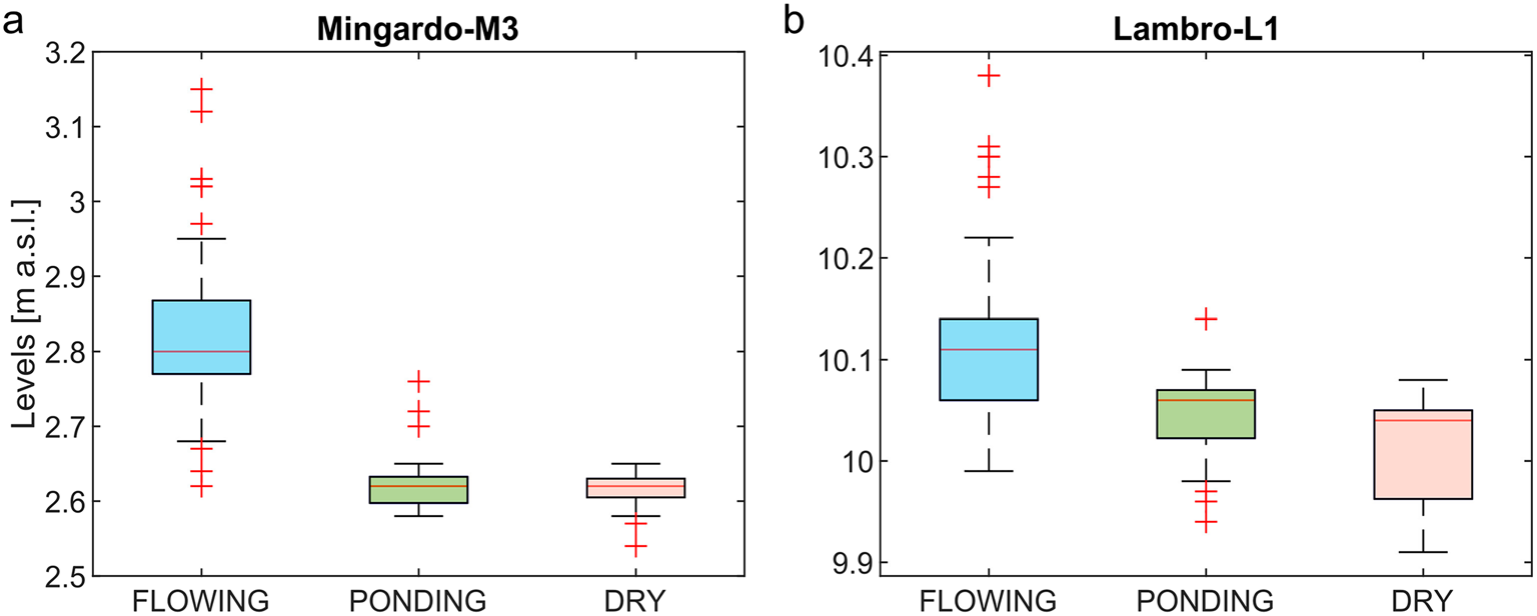
Distribution of water levels for considered flowing status: Flowing, Ponding and Dry. (**a**) M3 reach, (**b**) L1 reach.

In this paragraph is shown a comparison between the classification of flowing status obtained only from the FCIs analysis and the water level measurements available for M3 and L1 reaches. The daily water levels were recorded by two gauging stations, both located about 500 m downstream of reach M3 and L1 respectively. Figure 7a,b show the distribution of water levels for considered flowing status (Flowing, Ponding and Dry) for M3 and L1 reaches.

For the M3 reach of the Mingardo River (Fig. 7a) it is observed that, despite small regions of superposition, the water level measurements could be used to distinguish the F phase from the NF one (P and D phases). The ANOVA test, conducted between the “Dry” and “Ponding” groups of the M3 reach, provided a p-value equal to 0.16. For L1 reach (Fig. 7b), the regions of superposition are present for all three flowing status, and in this case the water levels are less indicative of which status occurs along L1 reach.

### Random Forest results of oversampled models

Cross validation (i.e. out-of-bag estimate) of oversampled RF models showed very high predictive capabilities, slightly different depending on the binary classification model (F/NF or P/D). Overall, the F/NF models performed better compared to the P/D ones, with accuracy ranging from 0.94 to 0.98 (mean = 0.955) and TSS from 0.88 to 0.95 (mean = 0.91). For P/D models, accuracy and TSS exhibited slightly lower scores ranging from 0.82 to 0.92 (mean = 0.865) and from 0.64 to 0.84 (mean = 0.73) respectively. On the one hand, for F/NF models, specificity (range 0.94–1.0, mean = 0.983) generally resulted greater than sensitivity (range 0.89–0.96, mean = 0.927), except for S1 reach. On the other hand, specificity (ranging from 0.77 to 0.92, mean = 0.843) was assessed to be lower than sensitivity (ranging from 0.86 to 0.92, mean = 0.888) for the P/D models. Furthermore, global models (GMs) generally were found to be slightly less accurate in predicting the correct flowing status compared to the locally calibrated models (i.e., individual reach models). In particular, for F/NF models the GM exhibited the lowest scores in terms of accuracy (0.94) and TSS (0.88). Whereas the GM for P/D models performed better exclusively compared to the M3 model which, due to a reduced number of observations in the training dataset (44), resulted to be globally the least accurate (accuracy = 0.82 and TSS = 0.64).

Our analysis demonstrated that the developed RF models are able to predict a specific flowing status (flowing/ponding/dry) occurring at the daily scale in every river reach. This quite robust prediction was achieved only using spatially interpolated rainfall and air temperature data. As the oversampled RF models demonstrated higher performances, exclusively these models were finally considered for further use and presented in Table 4.

**Table 4:** Predictive variables and performances obtained for the oversampled RF models.

Binary model	Model name	Predictive variables	Accuracy	Sensitivity	Specificity	TSS
F/NF	GM	R90, T90_MAX, R10	0.94	0.90	0.98	0.88
	S1	T90_MEAN, R30, R10	0.95	0.96	0.94	0.90
	M1	R90, R10, T90_MAX	0.95	0.91	1.00	0.91
	M2	R90, T90_MAX, R10	0.95	0.89	1.00	0.89
	M3	R90, T90_MAX, R10	0.98	0.96	0.99	0.95
	L1	T90_MAX, R90, R10	0.96	0.94	0.99	0.93
P/D	GM	R90, R30, T30_MAX	0.84	0.87	0.81	0.67
	S1	T90_MEAN, R90, R30	0.92	0.92	0.92	0.84
	M3	R90, T30_MAX, R30	0.82	0.86	0.77	0.64
	L1	R90, R30, T90_MAX	0.88	0.90	0.87	0.77

Models variables are listed in terms of descending importance for classification result (from left to right). Binary models code: F/NF = non-flowing/flowing, P/D = dry/ponding. Predictive variables code: R90 = cumulative 90-days rainfall, R30 = cumulative 30-days rainfall, R10 = cumulative 10-days rainfall, T90_MAX = average of previous 90-days maximum air temperature, T30_MAX = average of previous 30-days maximum air temperature, T90_MEAN = average of previous 90-days mean air temperature.

Concerning selected variables, the cumulative 90-days rainfall (R90) resulted relevant in 90% of all models, and turned to be the most important predictor in 70% of them. Whereas R10 and R30 were found to be important predictors to distinguish between F/NF and P/D phases in 100% of models respectively. R30 was furthermore assessed as significant predictor for the F/NF model of S1 reach. T90_MAX exhibited high importance especially considering F/NF models in which is present in 5 of 6 cases (83% of F/NF models). On the other hand, T90_MEAN was established as predictive variable for both binary models of S1 reach, and T30_MAX resulted an important predictor for P/D models in GM and M3 reach.

### Duration of flowing status

**Figure 8:**
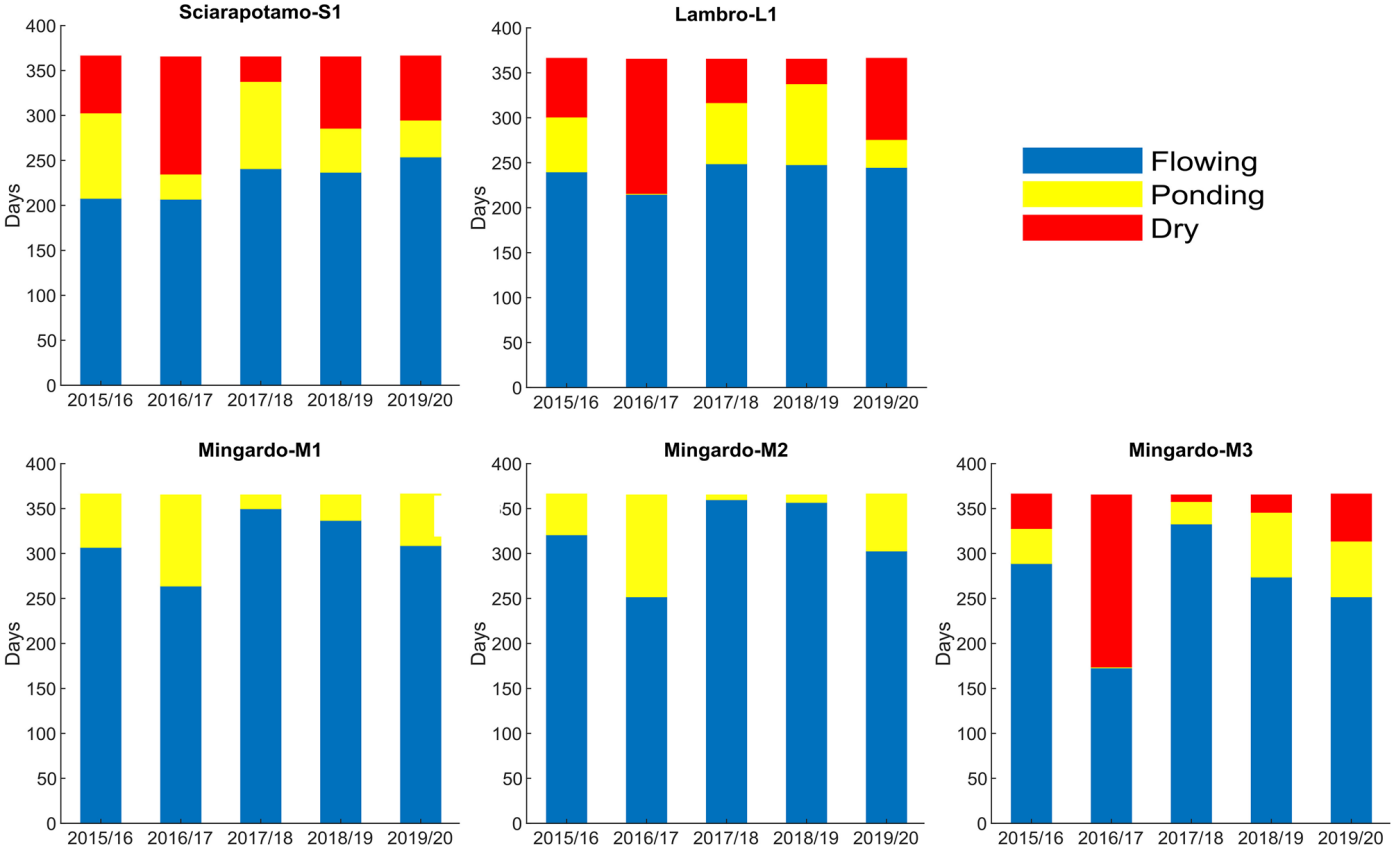
Daily occurrences of flowing (blue), ponding (yellow) and dry (red) phases predicted by the individual RF models for each reach during the study period from 2015 to 2020.

As the individual RF models demonstrated higher predictive capabilities, these models were than used to predict the duration daily frequency of each flowing status (F/P/D) in all considered reaches. In this way it was furthermore possible to determine the duration of F, P and D phases for each reach during the period 2015–2020 (Fig. 8).

Figure 8 shows that all the studied reaches are characterized by non-continuous water flow periods. In particular, for S1, L1 and M3 reaches, there is a period characterized by a completely dry river bed (D). Whereas for M1 and M2 reaches the dry phase never occurred, and, at non-flowing condition, isolated ponds of water are always present along these reaches (permanent ponding reaches).

For S1 and L1 reach, the average duration of NF was assessed to be about 4 months, and the dry bed period varied from a minimum of 27 days to a maximum of 149 days. For the other reaches, the yearly duration of the NF period has strong annual variations. For sections M1 and M2 the NF period varied between 5 and 113 days. For reach M3 the NF period was higher and with more pronounced variations (between 32 and 192 days). The duration of D period for M3 reach ranged between a minimum of 7 days to a maximum of 191 days. It is important to note that the obtained estimations of the yearly duration of the flowing status are affected by some uncertainty related to RF models performances. Particularly, uncertainty in the case of the drying reaches (S1, L1, M1) was higher, as misclassifications were produced by both F/NF and P/D models accuracy.

Overall, the longest duration of the ponding or dry-bed periods was observed in the year 2016/2017 for all the reaches studied. This longer duration is also reflected by the drought analysis conducted in Campania by Longobardi et al.^22^, which showed that a severe drought period occurred in 2017. The year with the shortest NF duration is the year 2017/2018 for S1, M1, M2, M3, while for L1 reach the years 2017/2018 and 2018/2019 have a similar duration of NF period, with the latter slightly shorter.

## Discussion

In this work, the Sentinel-2 dataset was exploited to monitor the flowing status of non-perennial rivers. Since the launch of the first of the two Sentinel-2 satellites (2015), 7 years of observations are available with a total dataset of 342 images for the observed area. Among these, 141 were actually exploitable, i.e. without cloud cover, with an effective revisit time of 15 days on average. The effective revisit time is reduced to 10 days on average in the dry season, from June to October, which is the most relevant period to observe the occurrence of ponding and dry phases.

The analysis of the spectral signatures of the land cover present in the non-perennial stream corridors revealed significant seasonal variations. Variation in water content of sediment bars and variation in water depth of the wet channel or ponds result in different reflectance values. This behavior makes it complicated to define an algorithm capable of distinguishing classes of sediment and water in a stable manner over time. However, spectral responses were sufficiently differentiated to distinguish the macro classes water, soil, and vegetation in a false-color image based on the B11, B8, and B4 bands.

This color combination makes it possible to identify the presence of water and distinguish the three flowing status. We have verified that this operates on several rivers from different geographical areas (e.g. Taro, Trebbia, Parma in northern Italy; Palancia River and Rambla de la Viuda in Spain). It follows that it is possible to apply the proposed method even when high-resolution images are not available and thus without repeating the analysis of spectral signatures. A classification with more classes, distinguishing for example, deeper pools from shallower pools or wet-sediment from dry-sediment may be possible. However, to further increase land cover classes, the spectral signatures should be derived from a much larger number of acquisitions and ground truths than the ones used in this study. In any case, the relationship between the spatial extent of the surfaces belonging to the different classes and the resolution of the satellite image is an important limiting factor. Our analyses demonstrated that the minimum width of a pond or wet channel that can be observed is variable depending on how the object is contained within the pixel. The comparisons with field measurements showed that it is possible to identify objects with a minimum width between 6 and 15 m. Consequently, more refined classifications with a larger number of classes are meaningful only for very wide riverbeds in which the different classes have sufficiently large sizes.

The FCIs are much clearer than images in true color (see Fig. 3) and allow to distinguish the phase of continuous water flow (F) from the one characterized by the presence of isolated ponds of water (P) and the dry bed phase (D) (see Fig. 4-5). This information can be used to define whether a river is non-perennial and, if so, to what degree, as proposed in many literature studies. For example, the four classes considered in the MIRAGE toolbox^38^: permanent, intermittent with pools in the non-flow period, intermittent with dry channel in the non-flow period, and episodic-ephemeral could be identified.

In the ponding phase it would be useful to identify the presence of pools, i.e. hydro-morphological units characterized by higher than average water depth. Combining the FCIs with an up-to-date DTM would provide such a distinction. Unfortunately, this approach is limited by the availability of DTMs with a frequency comparable to the rate of bedform change. An alternative possibility would be to observe on the FCIs the duration of water presence and identify pools as those where water presence persists longer.

The comparison between the punctual water level measurements, carried out with the traditional gauging systems and the satellite observations showed that the punctual measurements do not allow the distinction between the D and the P phases in both examined reaches (see Fig. 7). Moreover, for L1 reach it is not even possible to distinguish the F phase from the NF phase. Quite clearly, the water level at one point in a cross-section, is influenced by the local landscape, for example the presence of a pool or sediment deposit, and therefore cannot give information on the flow phase of the adjacent reach. This is particularly relevant during low flow phases. The considerations made show that even a greater, though desirable, spread of gauging stations would not allow appropriate identification of flowing status in non-perennial rivers and that satellite data are a crucial resource for this purpose.

Manual assignment of flowing status becomes time-consuming when the number of fluvial reaches to be observed is considerable. The results obtained can be used as baseline information to develop and calibrate an automatic algorithm for flowing status classification. The availability of such an algorithm will allow larger portions of the river network to be monitored.

The discontinuous observations of flowing status derived from Sentinel-2 images can be effectively exploited to calibrate models capable of making predictions on a daily basis. In this regard, the use of machine learning techniques (e.g., RF) which employ as predictor variables widely available hydrological data (such as rainfall or air temperature) resulted to be quite effective to this purpose. In particular, our approach, due to the very high predictive performances obtained for the developed RF models, can provide a valuable solution for assessing the flowing/ponding/dry duration in ungauged NPRs of Mediterranean region. In order to properly explore the relationship between the flowing status and the available data, the RF models were developed both at the local (individual reach, S1, M1, M2, M3 and L1) and global scale (all the reaches together, GM). Furthermore, we oriented our analysis developing binary classification RF models able to distinguish between both F/NF and the P/D phases. This two-step approach performed well, especially for distinguishing between the dry and the ponding phase, and was furthermore motivated in order to reduce class imbalance in the training dataset. Moreover it allows to proper consider the peculiarities of both permanent ponding and drying non-perennial river reaches. Indeed, a classification model with three classes (F/P/D) can be applied only in temporary rivers with the occurrence of all the three flowing status and may have lower performances compared to the presented approach (i.e., the 3-classes model accuracy were 0.84, 0.84 and 0.85, for L1, M3 and S1 reaches respectively, together with very low sensitivity values in the range 0.31–0.61 for the ponding class).

Among the predictive variables, it was observed that precipitation variables with higher temporal scales resulted to be of primary importance to correctly predict the flowing status in considered reaches. Indeed, the cumulative 90-days rainfall (R90) resulted relevant in 90% of all models. Only for F/NF model of S1 the most important precipitation predictor was assessed to be the cumulative 30-days rainfall (R30). Such result can be related to the specific characteristics of the considered reach, which mainly distinguishes itself due to the lowest drainage catchment extension (54 km^2^), and consequently to a lower time of concentration. R10 resulted as an important predictor for all F/NF models. In our opinion, this variable can embody the amount of cumulative rainfall required to generate a new flow phase after a period of non-flow in the considered reaches. On the other hand, the cumulative 30-days rainfall (R30) was found to be important for distinguishing between the occurrence of the ponding and dry phase. In this case, the R30 variables can be seen as a proxy of the groundwater level, therefore the grater the R30 value the higher the probability of being a day of ponding phase.

The implemented models take into account only precipitation and air temperature predictors and neglects many others that are significant in governing intermittency, such as catchment area, shape, orientation, slope, water table fluctuations and water seepage through riverbed. This is probably the reason why locally calibrated models are more accurate. However, in the study cases, also the global model showed high performances, but this may be due to the fact that considered rivers have similar hydro-climatic and geo-morphological characteristics. The availability of satellite observations makes it possible to obtain data for model calibration at the river reach scale. This allows to overcome the limitation of global models that can hardly describe the strong heterogeneity of the processes that determine a low spatial correlation of intermittence patterns as also observed by Snelder et al.^7^.

Once the Random Forest model has been calibrated in the period in which satellite images are available, it is possible to extend in the past the analysis of the hydrological regime to the entire time series of precipitation and temperature. However, it is important to consider that the model is calibrated against the actual presence of water in recent years so it cannot be applied to simulate the regime in previous periods if the basin conditions or water withdrawals have changed.

Recently, RF models were used by Messager et al.^1^ to simulate the natural regime of rivers across the globe. This approach does not allow to reproduce the actual regimes, i.e. those impacted by withdrawals and reservoirs, and consequently may underestimate the total number of non-perennial rivers. Likewise, hydrologic models calibrated against long historical data fail to model the actual river regime when changes related to climate, land use, reservoirs regulation and withdrawals occurred. In contrast, the approach developed in this paper, being based on observations of the regime in recent years may allow to make a more robust classification of F/NF phases at the reach scale and can used to estimate the effect of anthropogenic pressures due to water withdrawals. The regimes observed in different reaches of the same region allow to understand the peculiar reaction of each reach to the same atmospheric forcing such as dry periods. For example, the extreme drought experienced in the year 2016/17 resulted in a longer "dry" period in many reaches while in others the bed did not dry completely.

## Conclusion

The work proposes a methodology to identify non-perennial rivers and to assess their degree of temporariness. The data used are Sentinel-2 multispectral satellite images, distributed free of charge by the European Space Agency. Sentinel-2 has a nominal revisit time of around 5 days, however due to the occasional cloud cover the actual revisit time in the 2015–2021 years of observation averaged 15 days.

Analysis of the spectral signatures of the water, sediment, and vegetation classes allowed the identification of bands in which the spectral differences between the three classes are greatest. In red (B4), green (B3) and blue (B2) bands, water and vegetation have similar reflectance but can be distinguished from sediment. In the B8 band of the NIR, water and vegetation are clearly distinguishable while sediments are usually not distinguishable from vegetation. In the SWIR, B11 and B12 allow to distinguish fairly well all the classes. The FCIs composed of the B11, B8 and B4 bands resulted to bring out the differences between the three classes particularly well.

With FCIs it is possible to identify the three flowing status: continuous flow, disconnected pools and ponds and dry bed. Comparison with very high-resolution images and field observations showed very good agreement between estimated and actual flowing status. The analyses showed that wet channel or ponds are distinguishable if they are larger than 6 or 15 m in size. The random alignment between the water feature and the pixel determines whether the lower or the higher limit of the two applies.

The set of flowing status derived from the FCIs was used to calibrate random forest models capable of predicting the flowing status on a daily basis. Two types of binary models were implemented, the first was used to distinguish between “flowing” and “non-flowing” phases and the second further detailed the "non-flowing" phase by separating between "ponding" and "dry" phases. Several rainfall and temperature variables were tested as model predictors. Among these, the cumulative rainfall (R90, R30, R10) and temperature (T90_MAX, T90_MEAN, T30_MAX) with higher temporal scales resulted in the most relevant predictors for considered RF models. Our analysis revealed that locally calibrated RF models generally performed better for assessing the duration of flowing status in NPRs, nevertheless Global Models can be developed for multiple rivers reaches characterized by kindred hydro-climatic contexts.

The daily series of flowing status allow the estimation of the degree of temporariness of rivers through, for example, the duration of the "dry" and the "non-flowing" periods. In the study reaches of the Sciarapotamo and Lambro streams, the average duration of “non-flowing” resulted equal to about 4 months, with the “dry” period varying between 28 and 149 days. The upstream reach of the Mingardo stream resulted never “dry” and the “non-flowing” period ranged between 5 and 113 days. For the downstream Mingardo reach, the “non-flowing” period ranged from 32 and 192 days and the “dry” one was between 7 and 191 days.

The methodology developed in this work can make a crucial contribution to the identification and mapping water presence in non-perennial rivers and the estimation of the frequency and duration of each flowing status (flowing, ponding, dry). The free and global coverage of the source data, Sentinel-2 satellite mission imagery, and the easy-to-use method, may allow a cost-effective application in different river reaches worldwide.

## Supplementary Information

Supplementary Information 1.

Supplementary Information 2.

## Data Availability

The datasets used and/or analysed during the current study available from the corresponding author on reasonable request.
