# Supplementary material for: Exploiting Sentinel-2 dataset to assess flow intermittency in non-perennial rivers
Source: Sci Rep. 2022 Dec 16;12:21756. doi: 10.1038/s41598-022-26034-z (PMC9758196; doi:10.1038/s41598-022-26034-z)
Supplement: Supplementary file 1 — Supplementary Information 1. [file 41598_2022_26034_MOESM1_ESM.docx]

***Supplementary material***

Table 1 shows the classification of the flowing status for each river reach performed exploring the available Sentinel-2 images, over the period 2015-2021. Figure 1 shows some examples of how the various flowing status were assessed through visual interpretation of the false-colour images.

Table 1 Classification of flowing status for L1, M1, M2, M3 and S1 reaches from 2015 to 2021 and corresponding date of acquisition of the Sentinel-2 image

| Date | Reaches | | | | |
| --- | --- | --- | --- | --- | --- |
|  | L1 | M1 | M2 | M3 | S1 |
| 16/09/2015 | Dry bed | Ponding | Ponding | Ponding | Dry bed |
| 15/11/2015 | Ponding | Flowing | Flowing | Flowing | Ponding |
| 13/04/2016 | Flowing | Flowing | Flowing | Flowing | Flowing |
| 23/05/2016 | Flowing | Flowing | Flowing | Flowing | Flowing |
| 02/07/2016 | Ponding | Flowing | Flowing | Flowing | Ponding |
| 12/07/2016 | Ponding | Flowing | Flowing | Flowing | Ponding |
| 30/10/2016 | Flowing | Flowing | Flowing | Flowing | Flowing |
| 09/12/2016 | Flowing | Flowing | Flowing | Flowing | Ponding |
| 07/02/2017 | Flowing | Flowing | Flowing | Flowing | Flowing |
| 09/03/2017 | Flowing | Flowing | Flowing | Flowing | Flowing |
| 29/03/2017 | Flowing | Flowing | Flowing | Flowing | Flowing |
| 08/04/2017 | Flowing | Flowing | Flowing | Flowing | Flowing |
| 27/06/2017 | Dry bed | Ponding | Ponding | Dry bed | Dry bed |
| 07/07/2017 | Dry bed | Ponding | Ponding | Dry bed | Dry bed |
| 17/07/2017 | Dry bed | Ponding | Ponding | Dry bed | Dry bed |
| 06/08/2017 | Dry bed | Ponding | Ponding | Dry bed | Dry bed |
| 16/08/2017 | Dry bed | Ponding | Ponding | Dry bed | Dry bed |
| 26/08/2017 | Dry bed | Ponding | Ponding | Dry bed | Dry bed |
| 15/09/2017 | Dry bed | Flowing | Ponding | Dry bed | Ponding |
| 05/10/2017 | Dry bed | Ponding | Flowing | Dry bed | Dry bed |
| 15/10/2017 | Dry bed | Ponding | Flowing | Dry bed | Dry bed |
| 20/10/2017 | Dry bed | Ponding | Ponding | Dry bed | Dry bed |
| 25/10/2017 | Dry bed | Ponding | Ponding | Dry bed | Cloud Cover |
| 04/11/2017 | Dry bed | Ponding | Ponding | Dry bed | Ponding |
| 19/11/2017 | Flowing | Flowing | Flowing | Flowing | Flowing |
| 19/12/2017 | Flowing | Flowing | Flowing | Flowing | Flowing |
| 24/12/2017 | Flowing | Flowing | Flowing | Flowing | Flowing |
| 03/01/2018 | Flowing | Flowing | Flowing | Flowing | Flowing |
| 18/01/2018 | Flowing | Flowing | Flowing | Flowing | Flowing |
| 28/01/2018 | Flowing | Flowing | Flowing | Flowing | Flowing |
| 17/02/2018 | Flowing | Flowing | Flowing | Flowing | Flowing |
| 03/04/2018 | Flowing | Flowing | Flowing | Flowing | Flowing |
| 08/04/2018 | Flowing | Flowing | Flowing | Flowing | Flowing |
| 13/04/2018 | Flowing | Flowing | Flowing | Flowing | Flowing |
| 28/04/2018 | Flowing | Flowing | Flowing | Flowing | Flowing |
| 02/06/2018 | Flowing | Flowing | Flowing | Flowing | Flowing |
| 12/06/2018 | Flowing | Flowing | Flowing | Flowing | Ponding |
| 02/07/2018 | Ponding | Flowing | Flowing | Flowing | Ponding |
| 12/07/2018 | Ponding | Flowing | Flowing | Flowing | Ponding |
| 16/08/2018 | Ponding | Flowing | Flowing | Flowing | Flowing |
| 21/08/2018 | Flowing | Flowing | Flowing | Flowing | Flowing |
| 05/09/2018 | Ponding | Flowing | Flowing | Flowing | Ponding |
| 30/09/2018 | Dry bed | Flowing | Flowing | Ponding | Dry bed |
| 10/10/2018 | Ponding | Flowing | Flowing | Flowing | Ponding |
| 20/10/2018 | Ponding | Flowing | Flowing | Flowing | Dry bed |
| 25/10/2018 | Flowing | Flowing | Flowing | Flowing | Ponding |
| 30/10/2018 | Flowing | Flowing | Flowing | Flowing | Flowing |
| 14/11/2018 | Flowing | Flowing | Flowing | Flowing | Flowing |
| 29/11/2018 | Flowing | Flowing | Flowing | Flowing | Flowing |
| 19/12/2018 | Flowing | Flowing | Flowing | Flowing | Flowing |
| 07/02/2019 | Flowing | Flowing | Flowing | Flowing | Flowing |
| 17/02/2019 | Flowing | Flowing | Flowing | Flowing | Flowing |
| 27/02/2019 | Flowing | Flowing | Flowing | Flowing | Flowing |
| 09/03/2019 | Cloud Cover | Flowing | Cloud Cover | Cloud Cover | Flowing |
| 19/03/2019 | Cloud Cover | Flowing | Cloud Cover | Cloud Cover | Flowing |
| 24/03/2019 | Flowing | Flowing | Flowing | Flowing | Flowing |
| 18/04/2019 | Flowing | Flowing | Flowing | Flowing | Flowing |
| 07/06/2019 | Flowing | Flowing | Flowing | Flowing | Flowing |
| 12/06/2019 | Flowing | Flowing | Flowing | Flowing | Flowing |
| 27/06/2019 | Flowing | Flowing | Flowing | Flowing | Flowing |
| 02/07/2019 | Ponding | Flowing | Flowing | Flowing | Ponding |
| 17/07/2019 | Flowing | Flowing | Flowing | Flowing | Ponding |
| 22/07/2019 | Flowing | Flowing | Flowing | Flowing | Ponding |
| 27/07/2019 | Ponding | Flowing | Flowing | Ponding | Ponding |
| 06/08/2019 | Ponding | Flowing | Flowing | Ponding | Dry bed |
| 11/08/2019 | Ponding | Flowing | Flowing | Ponding | Dry bed |
| 21/08/2019 | Ponding | Flowing | Flowing | Ponding | Dry bed |
| 26/08/2019 | Ponding | Flowing | Flowing | Dry bed | Dry bed |
| 31/08/2019 | Ponding | Cloud Cover | Flowing | Dry bed | Dry bed |
| 05/09/2019 | Ponding | Cloud Cover | Flowing | Dry bed | Dry bed |
| 15/09/2019 | Ponding | Flowing | Flowing | Ponding | Dry bed |
| 30/09/2019 | Ponding | Flowing | Flowing | Ponding | Cloud Cover |
| 10/10/2019 | Ponding | Flowing | Flowing | Flowing | Dry bed |
| 15/10/2019 | Ponding | Flowing | Flowing | Ponding | Cloud Cover |
| 20/10/2019 | Dry bed | Flowing | Flowing | Ponding | Ponding |
| 30/10/2019 | Dry bed | Flowing | Flowing | Ponding | Ponding |
| 04/12/2019 | Flowing | Flowing | Flowing | Flowing | Flowing |
| 14/12/2019 | Flowing | Flowing | Flowing | Flowing | Flowing |
| 24/12/2019 | Flowing | Flowing | Flowing | Flowing | Flowing |
| 03/01/2020 | Flowing | Flowing | Flowing | Flowing | Flowing |
| 08/01/2020 | Flowing | Flowing | Flowing | Flowing | Flowing |
| 13/01/2020 | Flowing | Flowing | Flowing | Flowing | Flowing |
| 23/01/2020 | Flowing | Flowing | Flowing | Flowing | Flowing |
| 07/02/2020 | Flowing | Flowing | Flowing | Flowing | Flowing |
| 17/02/2020 | Flowing | Flowing | Flowing | Flowing | Flowing |
| 22/02/2020 | Flowing | Flowing | Flowing | Flowing | Flowing |
| 27/02/2020 | Flowing | Flowing | Flowing | Flowing | Flowing |
| 13/03/2020 | Flowing | Flowing | Flowing | Flowing | Flowing |
| 02/04/2020 | Flowing | Flowing | Flowing | Flowing | Flowing |
| 07/04/2020 | Flowing | Flowing | Flowing | Flowing | Flowing |
| 12/04/2020 | Flowing | Flowing | Flowing | Flowing | Flowing |
| 17/04/2020 | Flowing | Flowing | Flowing | Flowing | Flowing |
| 27/04/2020 | Flowing | Flowing | Flowing | Flowing | Flowing |
| 07/05/2020 | Flowing | Flowing | Flowing | Flowing | Flowing |
| 22/05/2020 | Flowing | Flowing | Flowing | Flowing | Flowing |
| 26/06/2020 | Ponding | Flowing | Flowing | Ponding | Ponding |
| 01/07/2020 | Cloud Cover | Flowing | Flowing | Ponding | Ponding |
| 06/07/2020 | Dry bed | Flowing | Flowing | Ponding | Ponding |
| 21/07/2020 | Dry bed | Flowing | Flowing | Ponding | Dry bed |
| 26/07/2020 | Dry bed | Ponding | Ponding | Ponding | Dry bed |
| 10/08/2020 | Dry bed | Ponding | Ponding | Dry bed | Dry bed |
| 15/08/2020 | Dry bed | Ponding | Ponding | Dry bed | Dry bed |
| 20/08/2020 | Dry bed | Ponding | Ponding | Dry bed | Dry bed |
| 30/08/2020 | Dry bed | Ponding | Ponding | Dry bed | Dry bed |
| 04/09/2020 | Dry bed | Ponding | Ponding | Dry bed | Dry bed |
| 09/09/2020 | Dry bed | Ponding | Ponding | Dry bed | Dry bed |
| 14/09/2020 | Dry bed | Ponding | Ponding | Dry bed | Dry bed |
| 19/09/2020 | Dry bed | Ponding | Ponding | Dry bed | Dry bed |
| 04/10/2020 | Dry bed | Flowing | Flowing | Ponding | Ponding |
| 09/10/2020 | Ponding | Flowing | Flowing | Flowing | Flowing |
| 19/10/2020 | Flowing | Cloud Cover | Flowing | Flowing | Flowing |
| 08/11/2020 | Ponding | Flowing | Flowing | Flowing | Ponding |
| 18/11/2020 | Flowing | Flowing | Flowing | Flowing | Flowing |
| 13/12/2020 | Flowing | Flowing | Flowing | Flowing | Flowing |
| 23/12/2020 | Flowing | Flowing | Flowing | Flowing | Flowing |
| 27/01/2021 | Flowing | Flowing | Flowing | Flowing | Flowing |
| 06/02/2021 | Flowing | Flowing | Flowing | Flowing | Flowing |
| 16/02/2021 | Flowing | Flowing | Flowing | Flowing | Flowing |
| 21/02/2021 | Flowing | Flowing | Flowing | Flowing | Flowing |
| 26/02/2021 | Flowing | Flowing | Flowing | Flowing | Flowing |
| 03/03/2021 | Flowing | Flowing | Flowing | Flowing | Flowing |
| 28/03/2021 | Flowing | Flowing | Flowing | Flowing | Flowing |
| 17/05/2021 | Flowing | Flowing | Flowing | Flowing | Flowing |
| 22/05/2021 | Flowing | Flowing | Flowing | Flowing | Flowing |
| 26/06/2021 | Ponding | Flowing | Flowing | Flowing | Ponding |
| 01/07/2021 | Dry bed | Flowing | Flowing | Flowing | Ponding |
| 06/07/2021 | Dry bed | Flowing | Flowing | Flowing | Ponding |
| 21/07/2021 | Dry bed | Flowing | Flowing | Ponding | Ponding |
| 31/07/2021 | Dry bed | Ponding | Ponding | Ponding | Dry bed |
| 10/08/2021 | Dry bed | Ponding | Ponding | Ponding | Dry bed |
| 15/08/2021 | Dry bed | Ponding | Ponding | Dry bed | Dry bed |
| 20/08/2021 | Dry bed | Ponding | Ponding | Dry bed | Dry bed |
| 25/08/2021 | Dry bed | Ponding | Ponding | Dry bed | Dry bed |
| 30/08/2021 | Cloud Cover | Cloud Cover | Cloud Cover | Dry bed | Cloud Cover |
| 09/09/2021 | Dry bed | Ponding | Ponding | Dry bed | Dry bed |
| 19/09/2021 | Dry bed | Cloud Cover | Ponding | Ponding | Dry bed |
| 24/09/2021 | Dry bed | Ponding | Ponding | Ponding | Dry bed |
| 04/10/2021 | Dry bed | Ponding | Ponding | Ponding | Ponding |
| 09/10/2021 | Ponding | Ponding | Ponding | Ponding | Ponding |
| 19/10/2021 | Ponding | Cloud Cover | Ponding | Ponding | Ponding |
| 18/11/2021 | Flowing | Flowing | Flowing | Flowing | Flowing |


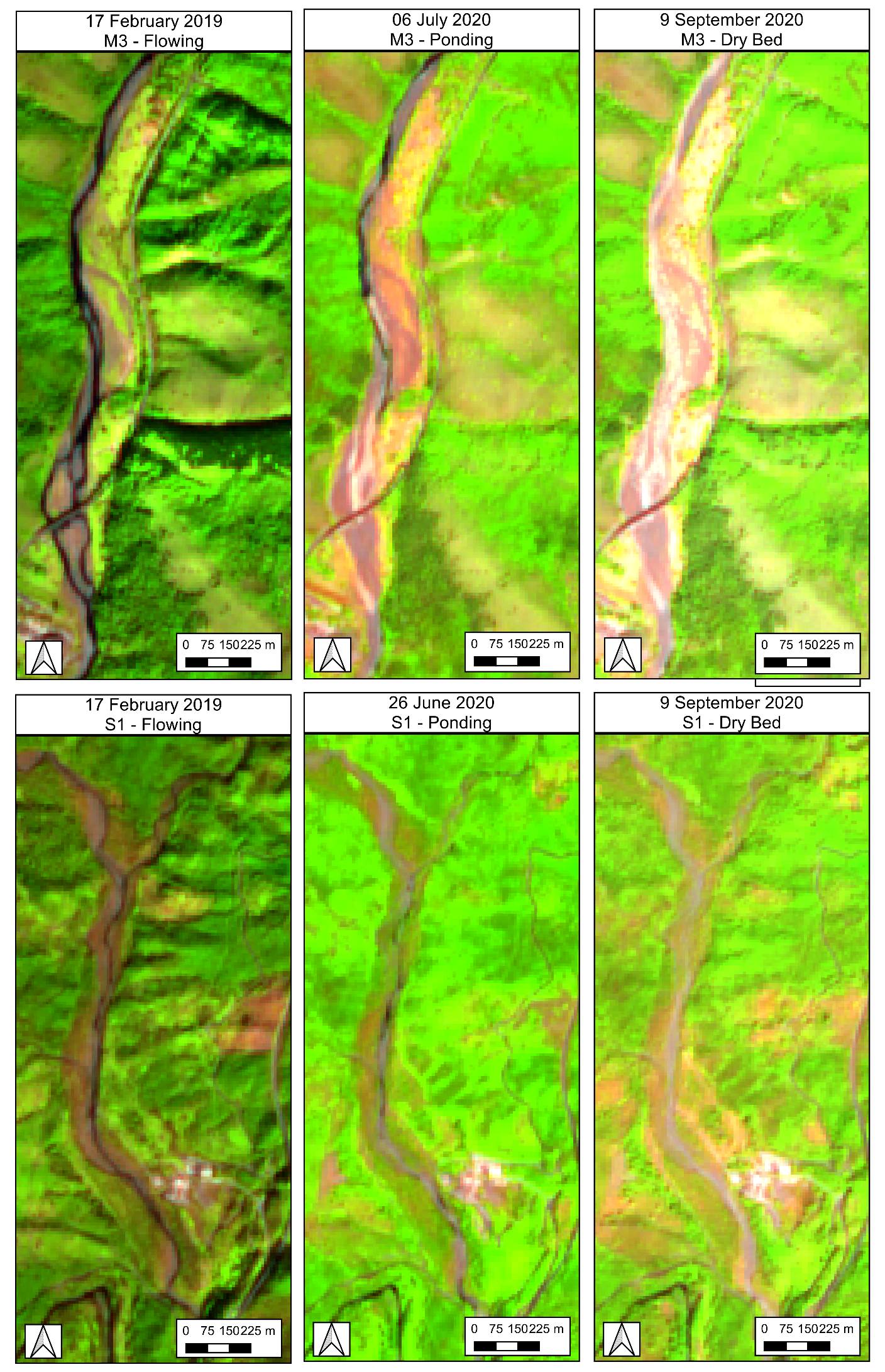


Figura 1: Flowing status of the 17 February 2019, 4 October 2020 and 9 September 2020, for M3 and S1 reaches.
