# Supplementary material for: Exploiting Sentinel-2 dataset to assess flow intermittency in non-perennial rivers
Source: Sci Rep. 2022 Dec 16;12:21756. doi: 10.1038/s41598-022-26034-z (PMC9758196; doi:10.1038/s41598-022-26034-z)
Supplement: Supplementary file 2 — Supplementary Information 2. [file 41598_2022_26034_MOESM2_ESM.docx]

**Step by step instruction to run FCI_maker tool**

This file describes the chain of commands to be executed to create the False Color Images (FCIs) composite of the SWIR, NIR and RED bands of Sentinel-2. The use of FCIs to observe the flowing status in non-perennial streams is shown in the paper “Exploiting sentinel-2 dataset to assess flow intermittency in non-perennial rivers” (under review). This explanation is intended for people who have no experience in using satellite data and the Google Earth Engine (GEE) platform.

To use the code, you need to create a Google Earth Engine account at the following link: <https://signup.earthengine.google.com/#!/>.

After registering on the GEE platform, you can access the FCI_maker code by selecting the following link: https://code.earthengine.google.com/220275e2e31d308773432fdecadc8a2f.

The FCI_maker code is divided into two parts:

The first part (from line 7 to line 23,) applies to the analysis of Sentinel-2, level-2A images, for which atmospheric correction has already been done. Level-2A Images are available from 2018 onwards, anyway depending on the study area, it is possible to find level-2A images even before 2018; the availability of level-2A images can be checked at https://scihub.copernicus.eu/dhus/#/home. It is advisable for images acquired in the year 2017 to first check whether these have been atmospherically corrected.

The second part of the code (from 31 line to 41 line,) applies to Level-1C images that are without atmospheric correction. For most applications it is always advisable to use atmospherically corrected images, however, in this particular case using images without atmospheric correction does not compromise the ability to visualize FCIs and observe the flowing status of stream flow.

To use the second part of the code (from 31 line to 41 line) it is necessary to insert the symbol "/*" at the beginning of line 7 and the symbol "*\" at the end of line 23. And remove the '/*' and '*\' symbols from lines 31 and 41.

The code is structured as follows:

lines 7 (or line 31): calling of the Sentinel-2 collection;

lines 10 (or line 32): application of a filter to get only images in the date of interest;

lines 13 (or line 33): application of a geographic filter to limit the search to images at the location of study area;

lines 19(or line 37): Zooming in on the study area (Optional);

lines 23 (or line 41): Creation of False Color images (FCI).

The user must perform the following steps:

1. Select the study area using the "Geometry imports" command, to do this:

- select "new layer”,
- select "Edit layer properties",
- assign the name “Selected_area”,
- choose the color (optional) and press ok,
- draw the boundaries of the study area and press exit.

2. Select the Sentinel-2 acquisition by entering in the command line 10 "filterDate" a date interval containing the date you want to display. Beware that the interval must be chosen so as to contain one and only one acquisition. Sentiel-2 acquisition dates can be searched at this page <https://scihub.copernicus.eu/dhus/#/home>. If in the chosen time interval there is no Sentinel-2 acquisition, the code will give you the following error: "Empty date ranges not supported for the current operation."

3. (Optional) if you want to center the resulting FCI image to a specific point of the map you can use the Map.setCenter command (line 17). In this case you need to remove the symbol "//" at the beginning of the line and to insert in the brackets the longitude of the center in degrees, the latitude of the center in degrees, the zoom level from 0 to 24);

4. click the "Run" button
